# Dietary, Metabolic, and Potentially Environmental Modulation of the Lysine Acetylation Machinery

**DOI:** 10.1155/2010/632739

**Published:** 2010-10-05

**Authors:** Go-Woon Kim, Goran Gocevski, Chao-Jung Wu, Xiang-Jiao Yang

**Affiliations:** ^1^The Rosalind & Morris Goodman Cancer Research Center, McGill University, Montréal, QC, Canada H3A 1A3; ^2^Department of Medicine, McGill University Health Center, McGill University, Montréal, QC, Canada H3A 1A3; ^3^Deparment of Anatomy & Cell Biology, McGill University, Montréal, QC, Canada H3A 1A3; ^4^Deparment of Biochemistry, McGill University, Montréal, QC, Canada H3A 1A3

## Abstract

Healthy lifestyles and environment produce a good state of health. A number of scientific studies support the notion that external stimuli regulate an individual's epigenomic profile. Epigenetic changes play a key role in defining gene expression patterns under both normal and pathological conditions. As a major posttranslational modification, lysine (K) acetylation has received much attention, owing largely to its significant effects on chromatin dynamics and other cellular processes across species. Lysine acetyltransferases and deacetylases, two opposing families of enzymes governing K-acetylation, have been intimately linked to cancer and other diseases. These enzymes have been pursued by vigorous efforts for therapeutic development in the past 15 years or so. Interestingly, certain dietary components have been found to modulate acetylation levels *in vivo*. Here we review dietary, metabolic, and environmental modulators of the K-acetylation machinery and discuss how they may be of potential value in the context of disease prevention.

## 1. Introduction

The link of lifestyles, such as dietary patterns and physical activity, to the risk of developing cancer and other diseases has received support from a plethora of epidemiological and biochemical studies. In line with this, a report from the World Health Organization (WHO) states that cancer causes 7.1 million deaths annually (12.5% of the global total) and dietary factors account for about 30% of all cancers in western countries and approximately up to 20% in developing countries (http://www.who.int/dietphysicalactivity/publications/facts/cancer/en/). The report also indicates that diet is second only to tobacco as a preventable cause. As developing countries become urbanized, not only does cancer incidence increase but also the patterns tend to shift towards those in economically developed countries. For example, the westernized diets and lifestyles (characterized by high calorie intake and insufficient physical activity), acquired in Japan in the past 60 years or so, are suspected to be a significant factor for increased incidence of breast, liver, colon, prostate, and pancreatic cancers in the country [1]. An important question is what are the mechanisms underlying the epidemiological shift? There may be multiple answers. One likely is that acquired diet and lifestyles might have altered or reprogrammed the epigenetic makeup of affected individuals, thereby contributing to the onset and development of cancer and other diseases. While a person's genetic blueprint remains relatively static, his or her epigenetic makeup is much more dynamic and thus susceptible to changes induced by different diet and/or lifestyle choices.

**Figure 1:**
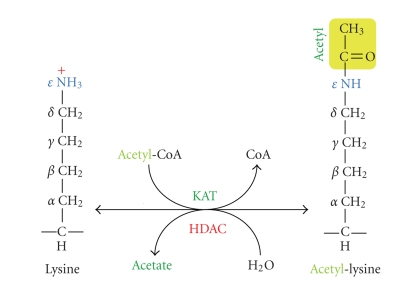
Cartoon illustrating acetylation and deacetylation at the *ε*-amino group of a lysine residue. A KAT is responsible for transfer of an acetyl moiety (in yellow) from acetyl-CoA to the *ε*-group of a lysine residue, whereas an HDAC removes the acetyl group from acetyl lysine, releasing acetate. Note that sirtuins use a catalytic mechanism that is completely different from what is illustrated here for the Rpd3/Hda1 family of deacetylases.

As a key component of the epigenetic makeup, lysine acetylation is now recognized as one fundamental posttranslational modification exerting profound effects on chromatin dynamics and other cellular processes in different species [2–4]. This modification transfers the acetyl moiety from acetyl-CoA to the *ε*-group of a lysine residue (Figure 1), which is reversible and dynamically governed by two groups of counteracting enzymes known as lysine acetyltransferases (KATs) and deacetylases (KDACs) [5–7]. Due to historical reasons, KDACs have almost exclusively been referred to HDACs (*h*istone *d*e*ac*etylases), so we keep the use of this acronym hereafter. Acetylation of specific lysine residues on histones is generally associated with transcriptional activation, whereas histone deacetylation results in transcriptional repression [8, 9]. However, exceptions to this general association have been reported. For example, some HDACs are involved in or associated with gene activation, and histone acetylation has been linked to assembly of heterochromatin [10–13]. 

In addition to histones, numerous nonhistone proteins with various functions in different organisms and distinct cellular compartments possess acetyl-lysine residues and are substrates of KATs and HDACs. On the rapidly growing list of acetylated proteins are transcriptional factors, metabolic enzymes, cytoskeleton regulators, chaperones, signaling modulators, and even viral and bacterial proteins [3, 14]. In agreement with this, the range of lysine acetylation substrates has been recently expanded further when proteome-wide experimentation identified thousands of K-acetylation proteins that are important for diverse cellular processes, which include chromatin remodeling, cell cycle progression, RNA splicing, mitochondrial metabolism, cytoskeleton dynamics, endocytosis, and vesicular trafficking [15–19]. Thus, regulation by K-acetylation not only operates at the level of gene expression but also acts through many other angles, including enzymatic activity or protein stability. 

**Figure 2:**
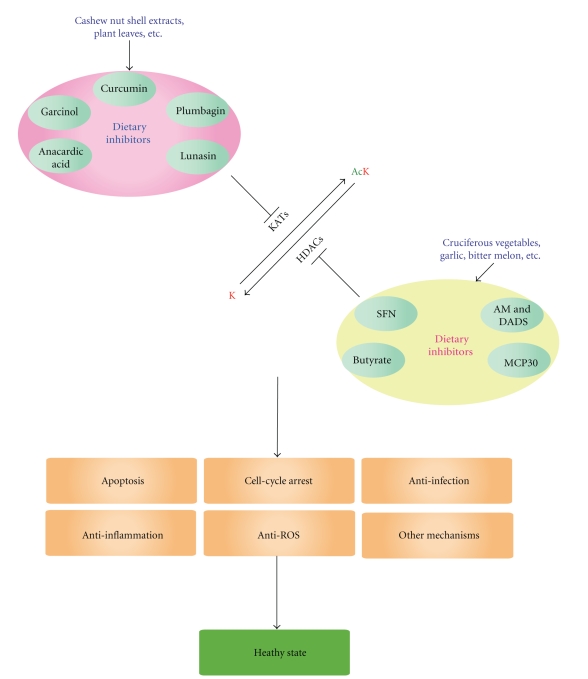
Diagram showing effects of dietary KAT and HDAC inhibition. The capital letter K (in red) refers to a lysine residue and Ac in green refers to acetylation. Different dietary components act on distinct KATs and HDACs to produce differential cellular effects depending on cell types and conditions. On the KAT and HDAC modulators illustrated here, all are small molecules except lunasin and MCP30, which are a 43-residue polypeptide and a 30 kDa protein, respectively. In addition to the inhibitors illustrated here, KAT and HDAC activators, as well as dietary patterns (such as high fat diet, high salt ingestion, and calorie restriction) and environmental factors, may target the acetylation machinery.

Delicate control is required for maintaining an appropriate acetylation profile for normal cellular functions. An imbalance has been associated with various diseases. As a result, many KAT and HDAC inhibitors, as well as activators, have emerged as promising agents for modulating this modification and treating diseases whose roots originate from altered K-acetylation. Several HDAC inhibitors have received approval from the US Food and Drug Administration (FDA) for treating cutaneous T-cell lymphoma [20]. In addition, scientists have discovered that some dietary components modulate KAT and HDAC activities (Figure 2), and have analyzed lifestyles to determine factors that may influence the functioning of these enzymes. Interestingly, endogenous molecules have also been shown to regulate the enzymatic activities. In what follows, we will list and review various natural and dietary modulators of KATs and HDACs (Figure 2), and briefly discuss how cellular metabolism, maternal diet, and environment may potentially affect K-acetylation *in vivo*.

## 2. Naturally Occurring KAT Inhibitors

### 2.1. Different Families of KATs

Since the very first KATs were identified in the mid-1990s, various mammalian proteins have been shown to possess such enzymatic activity [5, 21–23]. The three major groups are the GNAT (*G*cn5-related *N*-*a*cetyl*t*ransferases) superfamily, which includes HAT1 (*h*istone *a*cetyl*t*ransferase 1), GCN5 (*g*eneral *c*ontrol *n*on-derepressible 5), PCAF (*p*300/*C*BP-*a*ssociated *f*actor, paralog of GCN5), ELP3 (*el*ongation *p*rotein 3), ESCO1 (*es*tablishment of *co*hesion 1), ESCO2 (paralogous to ESCO1), and ATAC2 (*A*DA2A-con*ta*ining *c*omplex subunit 2); the MYST (*M*OZ, *Y*bf2/Sas3, *S*as2 and *T*ip60) family, which contains 5 members; and the p300/CBP family, composed of p300 (E1A-associated *p*rotein of 300 kDa) and CBP (*C*REB-*b*inding *p*rotein, paralogous to p300). A new and more systematic nomenclature system has proposed for these enzymes [23], but as a very recent addition to the GNAT family, ATAC2 needs to be incorporated into the system [24]. 

**Figure 3:**
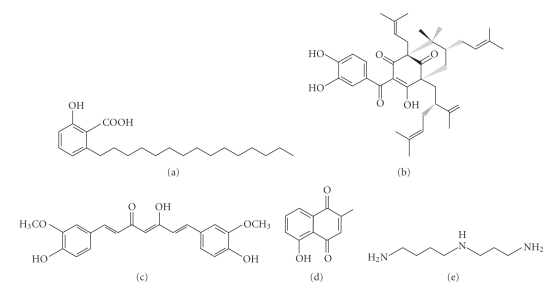
Chemical structure of naturally occurring KAT inhibitors. (a) Anacardic acid from cashew nut shell extracts; (b) garcinol from *Garcinia indica* fruit rind; (c) curcumin from *Curcuma longa* rhizome; (d) plumbagin from *Plumbago rosea* root extracts; and (e) spermidine as an endogenous molecule [28, 32, 33]. Some of these compounds have been modified to synthesize more enzyme-specific inhibitors, which are not depicted here [34, 35].

Both the GNAT and MYST families possess a homologous acetyl-CoA binding motif, have members in all eukaryotes, and are evolutionarily conserved from yeast to humans. One exception is ATAC2, which is only conserved from fly to humans although the slim mold and mushroom appear to contain remotely related proteins. The p300/CBP family does not display any sequence similarity to the GNAT and MYST families, and is only conserved from the worm to humans [25]; it is noteworthy, however, that the fungi-specific acetyltransferase Rtt109 is a structural homolog of p300 and CBP [26]. This pair of paralogs interacts with numerous binding partners and is indispensable in many critical cellular events [25]. As discussed below, they are frequent targets of known natural KAT inhibitors (Figures 2 and 3) although some of them also act on GCN5 and PCAF (see below) [27–29]. It is worth noting that little is known about the impact on other KATs, so this area remains to be developed. Moreover, only a few KAT activators have been identified despite a logical assumption that their potential therapeutic value can be as remarkable based on the wide use and established value of HDAC inhibitors.

### 2.2. Anacardic Acid

This is the first naturally occurring p300 inhibitor identified in 2003 (Figure 3(a)) [30]. It can be isolated from cashew nut shell extracts. A recent report stated that anacardic acid also inhibits the activity of Gcn5 from the malaria parasite *Plasmodium falciparum* and reduces growth of both drug-sensitive and -resistant strains [31]. With 271 genes in late trophozoites affected by anacardic acid treatment, 207 of them were down-regulated and associated with hypoacetylation of histone H3 at lysine 9 and 14 [31]. This compound possesses antitumor properties and inhibits p300-dependent transcription from chromatin templates but not from naked DNA templates [30].

### 2.3. Garcinol

It is a polyisoprenylated benzophenone derivative (Figure 3(b)) from the rind of the fruit-free *Garcinia indica* [32]. Garcinol has been shown to possess both antioxidant and anticancer chemopreventive activities in HeLa cells [32], and induces apoptosis in human leukemia cell lines [36]. It affects expression of numerous genes with oncogenic activities implicated in important cellular processes such as apoptosis and cell cycle regulation [32], suggesting potential value as anticancer therapeutics. As shown for anacardic acid, garcinol inhibits p300-dependent transcription from chromatin templates but not from naked DNA templates, suggesting chromatin- or histone-dependent action. Compared to anacardic acid, the advantage of garcinol lies in its ability to easily permeate the cellular membrane [30, 32]. In addition, several anacardic acid- and garcinol-based synthetic compounds have exhibited more specific effects on KATs [30, 34, 35], thereby encouraging development of related inhibitors with higher specificity and efficacy.

### 2.4. Curcumin

Also known as diferuloylmethane, curcumin is a polyphenol compound (Figure 3(c)) originating from the plant *Curcuma longa. *In addition to its use in traditional Indian and Chinese medicine as a therapeutic agent, it is often used in cuisine as a dietary spice and/or coloring agent [37]. One advantage of curcumin over the aforementioned two inhibitors is that it is a more specific inhibitor of p300 and CBP both *in vitro *and* in vivo.* Interestingly enough, curcumin possesses both antioxidant and prooxidant activities, which are thought to be the cause of its anticancer properties. At low concentrations, curcumin diminishes the formation of reactive oxygen species (ROS), whereas the opposite phenomenon is observed at higher concentrations [38]. Curcumin-induced anticancer effects have been by far extensively studied and demonstrated its efficacy in treatment of various diseases including colon [39] and ovarian cancers [40], metabolic diseases [41], and cardiac diseases [42]. Curcumin was also found to induce p53 acetylation and cause apoptosis [43]. Additionally, curcumin promotes apoptosis in brain cancer cells in a PARP- and caspase 3-dependent manner by inhibiting histone acetylation [37]. Moreover, both *in vitro* and *in vivo* approaches revealed a neurogenic activity of curcumin, so one potential avenue for the use of curcumin could be in replenishing some of the lost neurons that patients suffering from cerebral trauma experience [37]. Furthermore, HIV patients can also receive benefits from curcumin as it inhibits HIV multiplication by inhibiting acetylation of key viral proteins such as Tat and integrase [44]. Speaking of antiinfection, curcumin appears to possess antiparasitic activity towards *P. falciparum* [45, 46].

### 2.5. Plumbagin

Isolated from *Plumbago rosea* root extracts, plumbagin was found to potently inhibit KAT activity of p300 *in vivo* [33]. Plumbagin is a hydroxynaphthoquinone (Figure 3(d)), known in Indian ayurvedic medicine as Chitraka [33]. Interestingly, while most KAT inhibitors have polyhydroxyl functional groups, plumbagin has a single hydroxyl group indispensable for the inhibitory activity on p300. Substitution of this hydroxyl group with any other functional groups results in complete loss of the ability to inhibit p300 [33]. Plumbagin is highly cell-permeable and influences crucial cellular events. For example, it potently induces apoptosis at a high concentration partially through the NF-*κ*B pathway [47]. Since p300-mediated acetylation is required for NF-*κ*B activation, specific inhibition of p300 prevents the activation [33, 47]. In addition to histones, inhibition of p300 affects other nonhistone protein acetylation [33]. Emerging data also support biological relevance of this compound in neuroprotective, antibacterial, and anticarcinogenic activities [48–50], although inhibitory roles of plumbagin towards KATs in these cases remain to be investigated. In this way, plumbagin appears to be a potential useful agent for the prevention and treatment of various diseases, although plumbagin's high cellular toxicity limits its therapeutic applications [33]. Future development of synthetic analogues with lower toxicity shall circumvent this problem.

### 2.6. Lunasin

While the aforementioned 4 inhibitors are small molecules (Figures 3(a)–3(d)), lunasin is a 43-residue polypeptide. Derived from soy, barley, and wheat, luasin has cancer-chemopreventive value [51]. It has also been isolated from *Solanum nigrum* L., a plant that is indigenous to northeast Asia and has been traditionally used in oriental medicine [52, 53]. It was found that lunasin substantially inhibits activities of yeast Gcn5 and mammalian PCAF, resulting in decreased histone H3 and H4 acetylation [51–53]. Decrease in histone acetylation was perceived to occur simultaneously with inhibition of RB phosphorylation [51]. The binary effects on inhibition of histone acetylation and Rb phosphorylation by lunasin result in core histone hypoacetylation and cell cycle arrest, which will consequently render the cells abnormal, inhibit cell growth, and/or induce cell death. Oral administration is the main route for its cancer-preventive effects, so it is important to ensure that lunasin is absorbed and remains intact when reaching the site of action [51].

### 2.7. Spermidine

The intracellular concentration of spermidine, a natural polyamine (Figure 3(e)), decreases with aging [54]. Interestingly, a recent study found that spermidine promotes longevity [55]. Several organisms including yeast, flies, worms, mice, and human cells exhibit antiaging effects upon treatment with spermidine. Furthermore, an autophage-dependent mechanism seems to be at the origin for the prolonged life span. In addition, spermidine-mediated induction of longevity was associated with hypoacetylation at several lysine residues located at the N-terminal part of histone H3 and the spermidine-mediated anti-ageing effect phenocopied the knockout of KATs [55]. Although these results were produced by exogenous treatment of spermidine, considering that its concentration decreases with ageing and the KAT knockout phenocopies the stimulatory effects of spermidine in lifespan, it may function as an endogenous KAT inhibitor to regulate gene expression program in ageing.

## 3. Dietary and Endogenous HDAC Inhibitors

### 3.1. Two Different Families of HDACs

**Figure 4:**
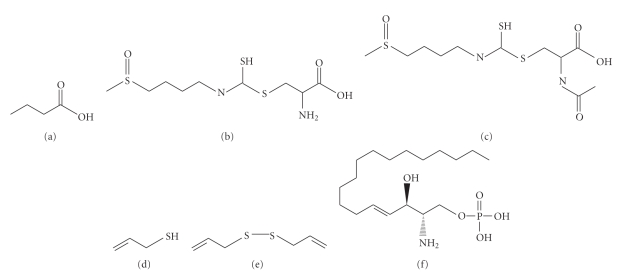
Chemical structure of dietary and endogenous inhibitors of HDACs. (a) butyric acid, acid form of butyrate, a product from dietary fiber fermentation by gut bacteria; (b, c) sulforaphane cysteine and sulforaphane *N*-acetyl-cysteine from cruciferous vegetables; (d, e) allyl mercaptan and diallyl disulfide from garlic; and (f) sphingosine-1-phosphate [62–64]. These compounds are either naturally or endogenously occurring, and they act on different members of the Rpd3/Hda1 family. The structures may be used as templates to generate more specific compounds with higher specificity and efficacy.

Compared to KATs, HDACs have been much more vigorously pursued as drug targets for cancer and other diseases. This is at least in part due to the fact that the first specific inhibitor trichostatin A was found to inhibit both cell proliferation and HDAC activity [56]. In humans, there are 18 known HDACs, which are divided into two families, based on sequence similarity to yeast orthologs, and catalytic mechanisms [57, 58]. The first 11 members, HDAC1–11, belong to the Rpd3 (*r*educed *p*otassium *d*ependency *3*)/Hda1 (*h*istone *d*e*a*cetylase *1*) family and are further grouped into three classes: I (HDAC1, 2, 3 and 8), II (HDAC4, 5, 6, 7, 9 and 10), and IV (HDAC11) [59]. The remaining seven are members of the Sir2 (*s*ilent *i*nformation *r*egulator *2*) or sirtuin (*Si*r*2*-related prote*in*) family and thus known as sirtuins 1–7 (or SIRT1–7) [60, 61]. They have also been referred to class III. While the Rpd3/Hda1 family members are Zn^2+^-dependent enzymes, sirtuins require NAD^+^ as a cofactor. Because of this, the modulators can be divided into two different groups according to which family of HDACs is the target. Most known inhibitors, including those listed in Figure 4 and discussed below, act on the Rpd3/Hda1 family.

### 3.2. Butyrate

Fermentation of dietary fibers by colonic bacteria produces several short chain fatty acids as end products, one of which is butyrate (Figure 4(a)) [65]. This is one reason for beneficial effects of dietary fibers. Butyrate was the first HDAC inhibitor identified in the late 1970s [66–68], and it might also be the smallest HDAC inhibitor known [62]. It affects diverse cellular processes, including gene expression, DNA synthesis, cell proliferation, and morphology [69]. Butyrate has a simple structure containing three carbons attached to a carboxylic acid group (Figure 4(a)), which is presumed to fit into the active site of HDACs and forms a bidentate ligand with zinc^2+^ buried there [70, 71]. Butyrate inhibits activities of most members of the Rpd3/Hda1 family, but not HDAC6 and HDAC10 [72]. 

In the context of cancer, it was observed that butyrate induces apoptosis through the action of PKC-*δ* and caspase 3, as well as suppresses survival effects of secondary bile acids in colon adenoma and cancer cell lines [65]. In another study, butyrate was shown to inhibit the recruitment of HDAC to the p21 promoter, thereby inducing the expression of p21 and concomitantly retarding cell proliferation [72]. Inhibition of HDAC activity by butyrate increases histone acetylation levels mediated by p300 at the promoter regions, leading to chromatin opening, p21 gene expression, and inhibition of E-Cdk2, which causes cell cycle arrest [72]. Apart from butyrate's anticancer capabilities, a mounting body of evidence points to multiple anti-inflammation roles: it can induce neutrophil apoptosis during systemic inflammation by activating the caspase cascade [73]; in neurodegenerative diseases such as spinal and bulbar muscular atrophy (SBMA), amelioration was seen after the oral administration of butyrate [74]; in the immune response butyrate inhibits IL-12 and TNF-*α* expression in monocytes [75], and in enteric neuroplasticity which is associated with increased histone H3 acetylation at lysine 9 [76]. 

Interestingly, a recent report showed that tributyrin, a prodrug of butyrate present in milk fat and honey, possesses more potential pharmacokinetic properties and is better tolerated orally than butyrate itself [77].

### 3.3. Sulforaphane (SFN)

An isothiocyanate (Figures 4(b) and 4(c)), SFN is derived from glucoraphanin and rich in cruciferous vegetables such as broccoli and broccoli sprouts. It was initially found as a potent inducer of phase 2 detoxification enzymes. However, the implication of SFN in inhibition of HDAC activity has gained particular attention [78]. Like other isothiocyanates, SFN is generated via the mercapturic acid pathway [79]. As the metabolized products of this pathway, both SFN-cysteine (SFN-Cys) and SFN-*N*-acetylcysteine (SFN-NAC) function as active HDAC inhibitors [80]. Compared to other HDAC inhibitors such as trichostatin A and SAHA (which act efficiently at nanomolar to low micromolar concentrations), SFN is a weak dietary inhibitor that requires a higher concentration to function [62, 71]. A pilot study has been performed to document the inhibitory effects of SFN on HDACs *in vivo*. In healthy volunteers ranging from 16–55 years of age without any history of nonnutritional supplement use, an intake of 68 g of broccoli sprouts with a bagel and cream cheese after 48 hours refraining from cruciferous vegetable consumption inhibited HDAC activity in peripheral blood mononuclear cells as early as 3 hours after consumption of the broccoli sprout [63]. The inhibitory effect of SFN on HDACs was also detected in human breast cancer cells [79, 81]. SFN showed anti-cancer properties by inhibiting HDAC activity, stimulating expression of proteins responsible for breast cancer cell proliferation and cell growth, and activating cell cycle arrest and apoptosis [81]. Furthermore, the study showed a significant reduction in expression of GFR, HER-2 and ER, the markers for breast cancer proliferation and clinical diagnosis. The induction of apoptosis in human breast cancer cell lines by SFN suggests that the caspase pathway might be involved. Future elucidation is required to examine the ability of SFN to inhibit HDAC activity on specific promoters since significant changes in global acetylation were not detected [81]. 

In prostate cancer, SFN treatment inhibited HDAC6, resulting in hyperacetylation of HSP90, disruption of the HSP90-androgen receptor (AR) interaction, and consequently, proteolytic degradation of AR [82]. Targeting AR by SFN is a therapeutically viable strategy for the treatment of prostate cancer since AR is the central protein in prostate cancer signaling. In line with this, AR's inactivation has been demonstrated as a promising strategy in preclinical studies and clinical trials [82]. Synergistic effects of SFN with polyphenol [-] epigallocatechin-3-gallate (EGCG), a compound found in green tea, have also been observed in human colon carcinoma cells [83]. EGCG is known to suppress colonic tumorigenesis in animal models and in epidemiological studies as well as to induce senescence in leukemic cells [83]. When taken in combination, SFN and EGCG attenuated the cellular senescence induced by EGCG and synergistically activated AP-1, a redox-sensitive transcription factor that regulates gene expression in response to oxidative and electrophilic stresses [83]. This approach may open new therapeutic methodology that different dietary agents function cooperatively to fight off cancer cells. Another study has demonstrated that SFN induces expression of an antimicrobial peptide called human *β*-defensin-2 in intestinal epithelial cells, implying the use of SFN not only in cancer but also in prevention and combat of bowel disorders such as Crohn's disease [84].

### 3.4. Allyl Mercaptan

Among organosulfur compounds derived from garlic, allyl mercaptan (AM, Figure 4(d)) achieves the greatest HDAC inhibition *in vitro* [64]. The inhibitory effect of AM on HDACs was detected in a dose-dependent manner within 10 min of exposure. In HT29 human colon cancer cells, AM treatment induced p21 expression and recruitment of Sp3 and p53 to the promoter. This coincided with growth inhibition and cell cycle arrest in G_1_. Structural analysis of AM binding to HDAC8 revealed that AM fits into the catalytic center without steric hindrance and is predicted to make hydrophobic interactions with residues in the cleft of HDAC8. Moreover, the predicted driving force of AM binding to the HDAC8 active site was suggested to be strong by the remarkably low free energy (−120 kcal/mol) in the interaction between zinc from HDAC8 and sulfur from AM [64]. 

Another organosulfur compound, diallyl disulfide (DADS, Figure 4(e)) has pleiotropic effects on various cellular events by modulating HDAC inhibition. DADS is a natural organosulfur compound found in garlic and accounts for 40%–60% of the total lipid-soluble sulfide in garlic oil [85]. In human colon adenocarcinoma cell lines, DADS treatment showed induction of G_2_ cell cycle arrest and inhibition of cell proliferation [86]. In rodent models, DADS inhibits the initiation and promotion phases of chemically induced carcinogenesis in digestive tracts such as colon, kidney, and stomach. The anticarcinogenic effect of DADS was due to its ability in inhibiting HDACs and causing hyperacetylation and induction of p21^Waf1^ gene expression, concomitant with increase in acetylation levels of histone H4 and H3 [86].

### 3.5. MCP30

A 30 kDa protein extracted from seeds of the bitter melon *Momordica charantia*, designated as MCP30 (*M*omordica *c*harantia *p*rotein of 30 kDa), was recently found to inhibit HDAC1 activity and promote H3 and H4 acetylation in several neoplastic prostate cell lines [87]. MCP30 induces apoptosis in premalignant and malignant prostate cells. Furthermore, these cells are marked by activity upregulation of HDAC1, whose expression and activity is selectively inhibited upon treatment with MCP30 [87]. These effects of MCP30 are associated with derepressed expression of PTEN, whose inactivation is highly significant in development of prostate cancer, inhibition of Wnt signaling, and decreased expression profiles of proteins, such as c-*Myc* and cyclin D1, known to control cell-cycle progression and apoptosis [87].

### 3.6. Nitric Oxide (NO)

The first endogenous HDAC inhibitor to be identified is nitric oxide, which selectively inhibits the activity of HDAC2. NO is generated by stimulation of brain-derived neurotrophic factor (BDNF) signaling and it nitrosylates cysteine 262 and 274 [88]. Interestingly, it was found that S-nitrosylation of HDAC2 does not inhibit the enzymatic activity, but rather facilitates removal of HDAC2 from chromatin and results in hyperacetylation of histones at the promoters of genes involved in neuronal development, which in turn causes activation of gene expression [88]. In another study, S-nitrosylation of HDAC2 by NO donors was observed to impair the enzymatic function of HDAC2 in dystrophin-deficient MDX mice, suggesting a different mechanism by which NO mediates S-nitrosylation of HDAC2 [89]. NO may also interfere with the binding of HDAC to gene promoter regions in the nucleus and inhibit the HDAC enzymatic activity in cytoplasm [90]. Taken together, these studies demonstrate that when an altered HDAC activity or expression is observed, such as Duchenne muscular dystrophy [89], inhibition of HDAC2 in an NO-dependent manner could be an interesting avenue to explore for the treatment of related diseases.

### 3.7. Sphingosine-1-Phosphate (S1P)

Recent data showed that sphingosine-1-phosphate (Figure 4(f)) acts as an endogenous inhibitor of HDAC1 and 2 [91]. Inhibition of the enzymatic activity by S1P was comparable to that of a well-known HDAC inhibitor, trichostatin A [91]. Subsequent mass spectrometry analysis revealed that S1P sits in the active site of HDAC1 or 2 for inhibiting the catalytic activity. S1P and SphK2, one isoenzyme responsible for synthesis of S1P, are present in a transcriptional corepressor complex containing HDAC1 and 2 [91]. In addition, interaction between SphK2 and purified core histones was observed and correlated with acetylation of specific lysine residues on core histones [91]. As witnessed in the case of butyrate, many HDAC inhibitors induce expression of p21; similarly, overexpression of SphK2 results in lysine 9 hyperacetylation of histone H3 at the p21 promoter [91], suggesting that SphK2 suppresses HDAC activity and promotes histone acetylation and p21 expression. Importantly, the discovery of endogenous HDAC inhibitors such as NO and S1P points out a novel approach in targeting HDACs.

## 4. Dietary Sirtuin Activation and Metabolic Impact on K-Acetylation

### 4.1. Dietary Sirtuin Activation

A natural and polyphenolic phytoalexin, resveratrol is present in grape skins and red wines, and is known to possess phytoestrogenic and antioxidant properties [92]. In addition, resveratrol also exists in a number of fruits, including mulberry, bilberry, cranberry and blueberry, as well as in peanuts [93]. A previous study has shown that resveratrol possesses diverse biochemical and physiological properties with health benefits, such as longevity enhancement, cardioprotection, anti-aging, anti-cancer activity, anti-inflammation, and neuroprotection [92, 93]. Intake of resveratrol alone and in combination with genistein, a major isoflavone found in soy, suppressed prostate cancer development in the SV-40 Tag rat model [94]. Also, beneficial metabolic effects induced by resveratrol have been observed in mice, for example, increase in aerobic capacity (increased running time and oxygen consumption in muscle fibers) [95]. The increase was attributed to induction of gene involved in oxidative phosphorylation and mitochondrial biogenesis. PGC-1*α*, a gene involved in mitochondrial biogenesis and function [95], is partly responsible for the observed changes. It seems that decreased PGC-1*α* acetylation is the cause for an increase in its activity. Reduced acetylation in PGC-1*α* and its subsequent elevation in functional capability were attributed to SIRT1, which interacts with and deacetylates PGC-1*α* at multiple lysine sites [95]. SIRT1, a member of the class III sirtuin family, senses the cellular energy by rise in the NAD^+^/NADH ratio and stimulates fat utilization to prevent diet-induced metabolic diseases [96]. It has been suggested that resveratrol activates SIRT1 in part by mimicking physiological pathways that activates SIRT1 such as caloric restriction (CR) [96]. A number of reports proposed that resveratrol also increases longevity. In fact, resveratrol treatment increased longevity of several model organisms including yeast, worm, and fly via sirtuin-dependant mechanisms [97, 98]. Resveratrol was also shown to extend lifespan of a short-lived species of fish, *N. furzeri*, by nearly 60% although the action of sirtuins involved in this effect has not been assessed [99]. The roles of the sirtuin family have been implicated in CR-mediated life span extension as well. One of the factors responsible for ageing in yeast is accumulation of extrachromosomal rDNA circles [100]. A higher ratio of NAD^+^/NADH caused by CR triggers Sir2 activation [101] and activated Sir2 then proceeds to induce chromatin silencing at the rDNA loci, thereby repressing the production of toxic rDNA circles [102]. 

However, there seems to have arisen a schism in the role of resveratrol or sirtuin activators in extension of lifespan in mammals. Two groups reported that resveratrol activates SIRT1 *in vitro* in a fluorescent-labeling-dependent manner, raising a debate if resveratrol even activates the sirtuins and if resveratrol does so in indirect mechanisms [103, 104]. In addition, the ability of resveratrol to extend lifespan has been questioned: only slight extension of lifespan had been observed in worms treated with resveratrol independently of Sir2 and no lifespan extension had been seen in flies, thereby contradicting some early studies [105]. Also, CR and resveratrol may induce longevity through sirtuin-independent pathways in mammals [106]. It was reported that CR and Sir2 overexpression in a yeast strain did not often extend lifespan and exhibited an additive effect on lifespan extension of yeast, suggesting they work through different mechanisms [107]. Consistently, resveratrol treatment exhibited a similar increase in neuronal survival of both Sir2 homozygous null flies and flies with normal Sir2 levels, likely operating in a Sir2-independent mechanism [108]. It was also found that CR showed more extended lifespan in a Sir2 knockout yeast strain than in the wild-type strain [107]. Furthermore, the sirtuins sometimes may limit lifespan rather than extend it [106]. This was shown by experimental results where Sir2 reduced chronological lifespan in yeast during the period of CR [109], suggesting that Sir2, instead of anti-aging activity, might induce aging in at least some types of mammalian cells and tissues. Overall, this is an actively debated research area [110, 111], so additional studies will be needed to fully elucidate the underlying molecular mechanisms of action.

### 4.2. Metabolic Impact on K-Acetylation

Metabolic states are tightly linked to regulation of the K-acetylation machinery. During fasting and feeding, cellular energy and metabolic states are reflected in fluctuations in levels of NAD^+^ and acetyl-CoA [112]. Upon feeding, the levels of acetyl-CoA, coenzyme of KATs, increase (Figure 1). Increased KAT activity then causes changes in chromatin dynamics and leads to the development of a certain epigenetic profile [112]. On the other hand, fasting elevates cellular NAD^+^ levels and leads to activation of sirtuins. Activated sirtuins then target chromatin and induce histone hypoacetylation to regulate gene expression according to the fasting state. 

Some of these principles have been evaluated in animal models. Two years ago, a group generated transgenic mice overexpressing SIRT1 (SirBACO mice) in the pursuit of identifying metabolic benefits that stem from the modulation of sirtuins [113]. SirBACO mice demonstrated a decrease in food intake and locomotor activity on a standard diet. Furthermore, SirBACO mice displayed diabetes-preventive effects, such as increased energy efficiency, which protects them from insulin resistance and hyperglycemia when they were put on a high-fat diet [113]. The study reveals an underappreciated role of sirtuins as cellular metabolism regulators. By analogy, activation of SIRT1 by a specific SIRT1 activator called SRT1720 shows preventive effects in metabolic disorders induced by a high calorie intake such as increased insulin sensitivity, increased fat oxidation, and suppressed obesity [96]. 

It has been known for decades that a reduction of calorie intake significantly lowers the rate of aging in mammals and decreases the risk of many age-related diseases, such as cancer and neurodegeneration [114]. For example, CR induced attenuation of A*β* peptides in brain tissues from mice by activating SIRT1, thus preventing Alzheimer disease-type amyloid neuropathology [115]. CR also induces SIRT3 expression, which plays an important role in fatty-acid oxidation; mouse expressing no SIRT3 exhibited impaired fatty-acid oxidation as well as elevated accumulation of fatty-acid oxidation intermediates and triglycerides in liver during fasting [116]. This increase in SIRT3 expression led to deacetylation and activation of the fatty-acid oxidation enzyme named LCAD (long-chain acyl coenzyme A dehydrogenase), potentiating a link between CR-induced SIRT3 activation and fatty-acid oxidation. In line with this, SIRT3 is an important sirtuin responsible for change of acetylation during CR [117]. A proteomic-wide survey revealed that among 287 unique acetylated proteins from mouse liver, 165 of them (about 57%) are mitochondrial proteins [117]. Among 165 mitochondrial proteins, 72 proteins were identified as candidates changing in acetylation upon CR. In addition, numerous *Salmonella enterica* proteins in central metabolism are acetylated, and acetyl-lysine residues are present in many mitochondrial proteins of mammalian cells and liver [15, 17, 18], further suggesting an intimate link between lysine acetylation and metabolism. Thus, it is reasonable to speculate that feeding behaviors and many metabolites will be found to target and modulate the acetylation machinery.

## 5. Impact of lifestyles and Environment on the K-Acetylation Machinery

### 5.1. Effect of Lifestyle Choices on Acetylation

As important as what kind of food a person has, how much he/she takes also affects the person's epigenetic state. Changes in carbohydrate/fat ratio in diet were found to alter acetylation of histone H3 and H4 on the sucrose-isomaltase gene [118]. A high carbohydrate diet intake enhances SI gene expression with increased acetylation levels of histone H3 and H4 on the enhancer/promoter and transcription regions of the sucrose-isomaltase gene [118]. Similarly, high-salt diet was found to induce the expression of salt-inducible kinases, which inhibit the action of HDAC5 [119]. Other lifestyle choices are also important factors contributing to disease development through altered acetylation. For example, smoking was reported to alter expression of class I/II HDACs in heavy smokers [120]. It was also reported that decreased HDAC activity by cigarette smoking was associated with induction of authophagy in chronic obstructive pulmonary disease (COPD) [121]. Moreover, substance abuse has been linked to altered histone acetylation. Chronic consumption of alcohol increases global acetylation of hepatic proteins and may induce liver injury [122]. Cocaine-induced neural and behavioral plasticity has been linked to altered histone acetylation [123].

### 5.2. Impact of Emotional Well-Being on Acetylation

Interestingly, emotional and psychological conditions have been implicated with HDACs and thus HDAC inhibitors. Transient decrease with a subsequent increase in acetylation of histone H3 was observed in the nucleus accumbens of mice that have undergone chronic social defeat stress [124]. Persistent increase in H3 acetylation was associated with a decreased level of HDAC2 in the nucleus accumbens. Chronic stress followed by delivery of butyrate into the nucleus accumbens generated robust antidepressant-like effects validated in several behavioral assays. Supportively, investigation in pursuit of beneficial value of butyrate in treating traumatic brain injury (TBI) found that concurrent butyrate treatment with behavioral training such as the Morris water maze task improved learning and memory in brain-injured mice [125], suggesting a role of HDACs in brain function. Related to this, HDAC5 mediates behavioral adaptation to emotional stimuli [126].

### 5.3. Link to Maternal Diet and Care to Offsprings' Acetylation

There is evidence suggesting that the maternal diet regulates gene expression and contributes to disease development in offsprings. Prenatal maternal diet with supplement of methyl donors has been shown to be associated with a higher risk of asthma characterized by a greater airway allergic inflammation and IgE production in F1 and F2 progenies [127]. It is likely that these effects involve differential epigenetic regulations caused by the maternal diet, behavior, and other factors. Supporting this notion, it was demonstrated that chronic consumption of maternal high-fat diet results in increased triglycerides and nonalcoholic fatty liver diseases, which are accompanied by significant hyperacetylation of histone H3 in fetal hepatic tissue at lysine 14, 9, and 18 [128]. 

Interestingly, maternal care exerts profound impact on offsprings' gene expression and epigenetic states [129, 130]. It was recently found that the hippocampus of offsprings of the high and low pup licking/grooming (LG) and arched-back nursing (ABN) mothers exhibits different programming of the glucocorticoid receptor (GR) gene promoter. Different maternal behaviors produced difference in histone acetylation, DNA methylation and interaction of the *GR* promoter with the transcription factor NGFI-A (nerve growth factor-inducible protein A) [131]. In addition, childhood abuse was found to affect methylation of the *GR* promoter in the brains of analyzed human subjects [132]. As functional impact of DNA methylation is often linked to histone deacetylation, maternal care might also regulate acetylation profiles in the offsprings. Furthermore, maternal aging was positively correlated with acetylation of histone H4 at lysine 12 in murine oocytes, which may further exert impact on fertilization and embryo development [133]. Taken together, external factors such as maternal diet and care could change the epigenetic program during development *in utero *as well as postnatally, thereby linking maternal factors to the epigenomic profile of an offspring.

### 5.4. Impact of Environmental Toxins on Acetylation

In addition to social and maternal environment, physical environment may affect acetylation of an individual. Exposure to an organochlorine pesticide called dieldrin was recently reported to play a role in apoptotic cells death in dopaminergic neuronal cells [134]. Dieldrin treatment induced acetylation of histone H3 and H4, which was associated with altered proteasomal activity and accumulation of CBP [134]. Further studies are needed to extrapolate cell-based findings to humans, but one immediate implication is that exposure to environmental toxins like dieldrin may directly impact the acetylation machinery.

## 6. Concluding Remarks and Future Perspectives

Since it was initially discovered and chemically defined in the 1960s [135, 136], acetylation of lysine residues on histones has gained much attention due to its important role in regulating gene expression and other chromatin-templated nuclear processes. As discussed above, potential nutritional or dietary components act through the K-acetylation machinery. Various studies suggest that this is an emerging and promising area of translational research, but vigorous criteria and efforts are needed to firmly establish a potential link, for example, how specific the link might be, and how it might fit in the existing literature about the biochemistry and biology of related KATs and HDACs. For example, biochemical studies have firmly established that GCN5 could be part of different multiple protein complexes [5, 137], so an interesting question is which complex should be the target of a potential modulator. A similar question can be raised for modulators of HDAC1 and HDAC2, both of which are part of multiple protein complexes [58, 59]. This way, related studies from different angles will synergize to facilitate the learning process about these enzymes and their regulators.

In addition to histones, thousands of nonhistone proteins are also subject to K-acetylation [15–19], so an interesting research direction would be how the inhibitors and activators (Figure 2) may regulate acetylation of these nonhistone proteins. This modification crosstalks with other phosphorylation, methylation, ubiquitination, and other posttranslational modifications to regulate protein functions in a coordinated fashion [3]. Thus, it is fair to conclude that K-acetylation (Figure 1) has emerged as one major regulatory mechanism in diverse biological systems from bacteria to humans. This has naturally led to rapid expansion of the research field about the responsible enzymatic machinery composed of KATs and HDACs (Figure 1), and has also resulted in the development and discovery of many inhibitors and activators of these enzymes, including those discussed above (Figures 3 and 4). Owing to their diverse roles in different cellular process, modulation of KATs and HDACs by inhibitors or activators has shed novel light on drug development and will likely become an important way for treating a variety of diseases. 

Just like environmental factors, dietary KAT and HDAC modulators are examples of dynamic regulators, which upon chronic exposure can modulate the cellular K-acetylation profile and imprint an individual's epigenetic makeup that may be static enough to last throughout his/her lifetime. One important question is what quantity of food needs to be ingested for these KAT and HDAC modulators to reach a physiologically relevant concentration at which they can either activate or inhibit KATs and HDACs to elicit disease-preventive effects. In this respect, sodium butyrate has been shown to cause cell cycle arrest and apoptosis at a concentration achievable in the colon by fiber consumption [138]. A similar conclusion has been reached in a pilot study involving the use of SFN in healthy volunteers [63]. Foods in different combinations could also represent how dietary modulators of KATs and HDACs confront human diseases. In addition, a better understanding of the pharmacokinetic makeup will help quantify the amount of food required for one to ingest and reach a therapeutic concentration. Another interesting question is whether more natural KAT and HDAC inhibitors or activators present in food or dietary agents remain to be identified (Figure 2). If so, the yet-to-be-discovered KAT and HDAC modulators could be very interesting research subjects, as they will open new avenues for drug development. 

Besides synthetic and naturally occurring compounds, emerging evidence supports the existence of endogenous KAT and HDAC regulators (Figures 3 and 4) and yields new insights into the development of novel therapeutic strategies that can target KATs and HDACs more specifically. Whether more endogenous KAT and HDAC modulators remain to be identified is an important question awaiting further investigation.

Major diseases such as cancer, diabetes, and heart disorders are often chronic, so in addition to therapeutic approaches, prevention is an important strategy for combating and managing these diseases. One relevant question is how to develop effective plans. As discussed above, daily activities of an individual, including food intake, physical activity, emotional well-being, physical and social environment, and other factors all seem to impact the K-acetylation machinery. Since the modification crosstalks with various regulatory mechanisms, daily activities may in turn affect epigenetic and other biological regulations. As we have witnessed in the past two decades the explosive studies of KATs, HDACs and their potential therapeutic value in treating and preventing cancer and other diseases, future investment and discoveries in this and related research areas will continue to generate not only novel and rich scientific knowledge but also practical information on how we can carefully manage daily activities as a life-long project for preventing diseases and improving the quality of life in the long run.
